# Exposure of Domestic Cats to Distinct *Ehrlichia canis* TRP Genotypes

**DOI:** 10.3390/vetsci8120310

**Published:** 2021-12-07

**Authors:** Ísis Assis Braga, Isis Indaiara Gonçalves Granjeiro Taques, Estefânia Crivelatti Grontoski, Ingrid Savino de Oliveira Dias, Nathalia Assis Pereira, Dirceu Guilherme de Souza Ramos, Filipe Dantas-Torres, Daniel Moura de Aguiar

**Affiliations:** 1 Veterinary Medicine College, Basic Unit of Bioscience, Mineiros University Center, 22nd Street s/n, Mineiros 75833-130, Goiás, Brazil; isis@unifimes.edu.br; 2 Virology and Rickettsioses Laboratory, Department of Clinical Veterinary Medicine, Veterinary Medicine College, Federal University of Mato Grosso, Fernando Correa da Costa Avenue 2367, Cuiabá 78060-900, Mato Grosso, Brazil; isis_indaiara@hotmail.com (I.I.G.G.T.); estefania_grontoski@hotmail.com (E.C.G.); ingrid_savino1@hotmail.com (I.S.d.O.D.); nathaliaassis89@gmail.com (N.A.P.); 3 Laboratory of Veterinary Pathology and Parasitology, Academic Unit of Agricultural Sciences, Federal University of Jataí, Jataí 75801-615, Goiás, Brazil; dgramos_vet@hotmail.com; 4 Department of Immunology, Aggeu Magalhães Institute, Oswaldo Cruz Foundation, Professor Moraes Rego Avenue s/n, Recife 50670-420, Pernambuco, Brazil; filipe.torres@fiocruz.br

**Keywords:** ELISA, ehrlichiosis, feline, IFAT, serodiagnosis

## Abstract

Cats naturally exposed to *Ehrlichia canis* have been described in different regions of the world, but little is known about the genotypes associated with infection in these animals. To detect *E. canis*-specific antibodies and investigate the *E. canis* TRP genotypes in cats, serum samples from 76 domestic cats reactive to crude *E. canis* antigens by the indirect fluorescence antibody test (IFAT) were analyzed by ELISA, using *E. canis*-specific peptides (i.e., TRP19 and TRP36 /BR/US/CR). Of these, 25 (32.9%) cats reacted to at least one TRP peptide, confirming their specific exposure to *E. canis*. Eighteen (23.7%) cats reacted to TRP19, 15 (19.8%) to BRTRP36, and 11 (14.5%) to USTRP36, but none of them reacted to CRTRP36. Eight (10.5%) cats reacted to TRP19 but not to any TRP36 genotype, demonstrating the possible existence of a new *E. canis* genotype infecting felines. Nevertheless, this study provides the first report of anti-*E. canis*-specific antibodies in domestic cats.

## 1. Introduction

*Ehrlichia canis* infection has been increasingly detected in cats in many countries, using both molecular and serology techniques [1,2,3,4,5]. However, the body of knowledge about the etiological agents and pathogenesis of feline ehrlichiosis is still incomplete. The pathogenesis of canine monocytic ehrlichiosis (CME) is well characterized and it is known that during the acute or chronic phases of the disease, *E. canis* tends to remain at undetectable levels in the bloodstream, and that serological methods are useful for detecting carrier animals [6].

Molecular targets have been identified and used in the diagnosis of ehrlichiosis, and a group of proteins that have amino acid repeats (TRP—tandem repeat proteins) have proved to be important immunoreactive proteins of *E. canis*. TRP proteins are responsible for reprogramming the host cell and favoring the intracellular survival of bacteria [7]. The TRP19 protein is highly conserved among the *E. canis* isolates described so far, making it a specific target for the serological diagnosis of CME. Due to the particularities of their amino acid sequences, these proteins are useful for distinguishing anti-*E. canis* antibodies of other species of the genus [8,9,10,11,12].

The TRP36 protein demonstrates a high degree of diversity among several *E. canis* isolates [11,13,14]. To date, three genotypes based on the characteristics of this protein have been described: the American genotype (USTRP36) [9], the Brazilian genotype (BrTRP36) [13], and the Costa Rican genotype (CRTRP36) [14].

Given that dogs and cats have similar *E. canis* infection rates in some endemic areas in Brazil [4,15,16,17], it would be interesting to investigate the presence of anti-*E. canis* antibodies in cats using TRP19, and to examine the different TRP36 genotypes in these animals. Therefore, in view of the sensitivity and specificity of these proteins in the immunodiagnosis of ehrlichiosis [12,18], the purpose of this study was to identify anti-*E. canis* antibodies in cats using TRP19, and to distinguish the genotypes detected using synthetic peptides of three TRP36 proteins, based on enzyme-linked immunosorbent assays (ELISA).

## 2. Materials and Methods

### 2.1. Samples

A total of 76 serum samples from cats that underwent a previous indirect fluorescence antibody test (IFAT) using crude *E. canis* antigens (São Paulo strain) [4] were used in this study (titers above 40 were considered positive). In brief, these samples were obtained from both stray and domestic cats living in the metropolitan region of Cuiabá, state of Mato Grosso, as described elsewhere [4].

### 2.2. ELISA

All IFAT-positive samples were diluted at 1:200 to be used in ELISA. Anti-*E. canis* antibodies were evaluated against the peptide corresponding to the region of the epitope of protein *E. canis* TRP19 (HFTGPTFSEVNLSEEEKMELQEVS) [19]. To define the genotype responsible for the infection, we used immunoreactive peptides corresponding to the epitopes of the *E. canis* TRP36 tandem repeat (TR) regions of the American (USTRP36) (TEDSVSAPATEDSVSAPA) [9], Brazilian (ASVVPEAEASVVPEAEASVVPEAE) [12,13], and Costa Rican genotypes (EASVVPAAEAPQPAQQTEDEFFSDGIEA) [20]. The peptide corresponding to the C-terminal region of the TRP36 protein of the Israeli isolate of *E. canis* (IS36-C-V, NPTGLKFLDLYTQLTL) was used as a control peptide, due to its low immunoreactivity [11]. The peptides were commercially synthesized (AminoTech, Diadema, Brazil), resuspended in ultrapure water at a concentration of 1 mg/mL, and stored at −20 °C until the moment of analysis.

The assays were performed as described by Aguiar et al. [12]. The optical density (OD) of each tested serum was represented by the average of three readings (each peptide adsorbed in triplicate) subtracted from the OD of the control peptide (IS36-C-V). A cutoff value of ≥0.150 and gray zone between 0.135 and 0.149 were previously defined based on the ODs of 20 samples seronegative by IFAT [21].

## 3. Results

Of the 76 samples tested, 25 (32.9%) were reactive against at least one of the TRP peptides (Table 1), whereas 51 (67.1%) did not react to any of the peptides. 

**Figure 1:**
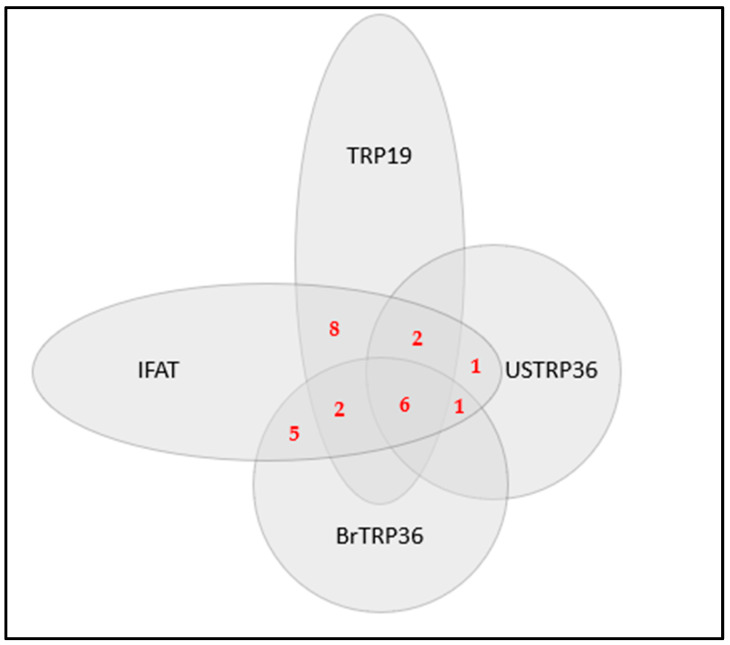
Venn diagram showing combinations of *Ehrlichia canis* antibodies detected in feline serum samples subjected to the indirect fluorescence antibody test (IFAT) with *Ehrlichia canis* antigens, and enzyme-linked immunosorbent assay (ELISA) with synthetic peptides TRP19, BrTRP36 and USTRP36.

Eighteen (23.7%) samples reacted to TRP19 (OD range, 0.172–1.481; OD average, 0.602), 14 (18.4%) to BrTRP36 (OD range, 0.166–0.501; OD average, 0.306), and 10 (13.2%) to USTRP36 (OD range, 0.159–1.184; OD average, 0.351), but none of the samples reacted to CRTRP36. The detection of more than one genotype in the same animal, or detection of a single genotype, is illustrated in the venn diagram in Figure 1.

## 4. Discussion

A previous study reported that domestic cats in central west Brazil are frequently exposed to *Ehrlichia* spp. [4]. In the present study, it was found that less than half of these animals (32.9%) reacted to at least one of the studied peptides, confirming their previous exposure to *E. canis*. Considering the specificity of the peptides in the serodiagnosis of *E. canis* [19], this study reports for the first time the presence of anti-*E. canis*-specific antibodies in domestic cats. It should be noted that all the animals had been naturally infected, and that not all had undergone a previous clinical analysis, thus making it impossible to predict the time elapsed from the onset of infection.

Most of the samples (67.1%) presented seronegative results in all the ELISA assays using synthetic peptides of *E. canis*, suggesting that some of the reactions observed by IFAT in cats are nonspecific. IFAT is considered the gold standard in the serological diagnosis of CME [6]. However, this technique has some disadvantages, such as cross-reactivity with antibodies produced against antigenically similar microorganisms (e.g., other species of the genus *Ehrlichia* and even members of the genus *Anaplasma*), possibly leading to misinterpretations of the results [18].

The TRP36 protein has been useful for evaluating the genetic and antigenic diversity of *E. canis* isolates. The samples tested in this study reacted to BrTRP36 (18.4%) and USTRP36 (13.2%) peptides, indicating that the Brazilian and American genotypes circulate among cats in central west Brazil. This finding corroborates the data presented by Aguiar et al. [13], who observed the presence of both genotypes in dogs in Brazil. Taques et al. [22] also reported serological reactions to the BrTRP36 and USTRP36 genotypes and described for the first time the finding of dogs reactive to CRTRP36 in Brazil. Recently, dogs in Colombia and Turkey were found to be infected by the three genotypes [20,23].

Cárdenas et al. [18] showed that infected dogs reacted earlier (14 days post-infection) to TRP36 than to TRP19 (21 days post-infection), which may explain our findings that 9.2% of samples reacted to the TRP36 peptide (Br and US), but not to TRP19. The duration of infection of the animals in this study was unknown, so we hypothesized that they were in different phases of infection. Cárdenas et al. [18] also demonstrated that ELISA using recombinant peptides (including TR19 and TR36) is highly sensitive, indicating its usefulness as a tool for the diagnosis of ehrlichiosis. Indeed, the high sensitivity and specificity of ELISA in detecting antibodies to *E. canis* recombinant peptides enable a more accurate and early diagnosis of the infection.

Our results suggest that domestic cats in Brazil could concomitantly harbor the Brazilian and American TRP36 genotypes. The presence of antibodies against both BrTRP36 and USTRP36 indicates that, like dogs, coinfection by the two genotypes may also occur in cats. As discussed previously [12,13], coinfection by different genotypes of *E. canis* may give rise to a recombination point between different proteins, which has also been observed in different isolates of *E. ruminantium* [24]. Such recombination may be involved in the agent’s ability to infect other mammalian hosts, in addition to dogs and *Rhipicephalus sanguineus* lato sensu ticks. A genotype having a yet unknown tandem repeat sequence of TRP36 protein may also be circulating among cats in Brazil, since 10.5% of the samples reacted to TRP19, but not to BrTRP36, USTRP36, or CRTRP36. This has also been observed in dogs [12]. In the present study, no reactions were observed against the CRTRP36 peptide. However, it should be noted that the feline positive control for this antigen was not included in the assays, which is a weakness of this investigation. As mentioned earlier, Taques et al. [22] found low frequencies of reactions to the CRTRP36 peptide against BR and US. In other words, it was a genotype detected infrequently among canines, hence the difficulty in achieving good control for felines. Regarding its reactivity, this peptide was previously validated as suitable for use in immunoenzymatic assays [20].

It is suspected that divergences in TRP36 protein are involved in the infection of different species of mammals [14,25]. While feline infections have been reported in Brazil since 1998 [26], it is reasonable to assume that the divergence of TRP36 in Brazil may also be associated with cats, since some felines were more reactive to BrTRP36 than to USTRP36.

## 5. Conclusions

This study confirms the involvement of *E. canis* in the etiology of *Ehrlichia* infection in cats in Brazil, highlighting the need to include feline ehrlichiosis in the differential diagnosis of vector-borne diseases in cats in this country. Furthermore, our study raises the hypothesis that a new *E. canis* TRP36 genotype may be circulating in domestic cats; thus, further investigation is needed.

## Figures and Tables

**Table 1:** Detection of *Ehrlichia canis* antibodies in feline serum samples subjected to the indirect fluorescence antibody test (IFAT) with *Ehrlichia canis* antigens, and enzyme-linked immunosorbent assay (ELISA) with synthetic peptides TRP19, BrTRP36 and USTRP36.

Sample	IFAT	ELISA (Optical Density)		
	Titer	TRP19	BrTRP36	USTRP36
#514	320	0.034	0.289	0.002
#803	160	0.308	0.004	0.016
#434F	1280	1.104	0.004	0.097
#11055	80	0.416	0.181	0.299
#1664	1280	0.050	0.501	0.133
#1831	160	0.331	0.200	0.163
#2156	160	1.104	−0.007	0.007
#2519	40,960	1.480	0.031	1.184
#2958	1280	0.210	0.330	0.147 †
#307	160	0.113	0.316	0.227
#347	160	0.328	0.166	0.159
#631F	640	0.052	0.183	0.052
#724	640	0.207	0.167	0.126
#8078	80	0.172	0.055	0.025
#93	1280	0.411	0.089	0.070
#9717	1280	1.481	0.025	0.013
#GT09	160	0.672	0.031	0.009
#GT13	80	0.036	0.042	0.251
#GT18	80	0.007	0.262	0.000
#I-14	160	0.800	0.390	0.197
#I-16	80	0.584	0.389	0.468
#I-31	640	0.621	0.483	0.311
#9	640	0.206	0.031	0.036
#134	640	0.405	0.121	0.247
#151	160	0.005	0.426	0.018

Underlined values were considered positive; † indeterminate (gray zone).

## Data Availability

The raw data have not been published or stored elsewhere, but are available upon request from D.M.d.A.
